# Microenvironmental adaptation of experimental tumours to chronic *vs* acute hypoxia

**DOI:** 10.1038/sj.bjc.6602066

**Published:** 2004-08-10

**Authors:** O Thews, T Wolloscheck, W Dillenburg, S Kraus, D K Kelleher, M A Konerding, P Vaupel

**Affiliations:** 1 Institute of Physiology and Pathophysiology, University of Mainz, Duesbergweg 6, 55099 Mainz, Germany; 2 Institute of Anatomy, University of Mainz, Becherweg 13, 55099 Mainz, Germany

**Keywords:** hypoxia, oxygenation, vascularity, perfusion, VEGF, cell proliferation

## Abstract

This study investigated long-term microenvironmental responses (oxygenation, perfusion, metabolic status, proliferation, vascular endothelial growth factor (VEGF) expression and vascularisation) to chronic hypoxia in experimental tumours. Experiments were performed using s.c.-implanted DS-sarcomas in rats. In order to induce more pronounced tumour hypoxia, one group of animals was housed in a hypoxic atmosphere (8% O_2_) for the whole period of tumour growth (chronic hypoxia). A second group was acutely exposed to inspiratory hypoxia for only 20 min prior to the measurements (acute hypoxia), whereas animals housed under normal atmospheric conditions served as controls. Acute hypoxia reduced the median oxygen partial pressure (*p*O_2_) dramatically (1 *vs* 10 mmHg in controls), whereas in chronically hypoxic tumours the *p*O_2_ was significantly improved (median *p*O_2_=4 mmHg), however not reaching the control level. These findings reflect the changes in tumour perfusion where acutely hypoxic tumours show a dramatic reduction of perfused tumour vessels (maybe the result of a simultaneous reduction in arterial blood pressure). In animals under chronic inspiratory hypoxia, the number of perfused vessels increased (compared to acute hypoxia), although the perfusion pattern found in control tumours was not reached. In the chronically hypoxic animals, tumour cell proliferation and tumour growth were significantly reduced, whereas no differences in VEGF expression and vascular density between these groups were observed. These results suggest that long-term adaptation of tumours to chronic hypoxia *in vivo*, while not affecting vascularity, does influence the functional status of the microvessels in favour of a more homogeneous perfusion.

Oxygen deficiency (hypoxia) can be found in many human malignancies (Vaupel *et al*, 1989; Höckel and Vaupel, 2001) and has been blamed for limiting the efficacy of several nonsurgical tumour treatments such as sparsely ionising radiation (Gray *et al*, 1953; Bush *et al*, 1978; Horsman, 1993), O_2_-dependent chemotherapeutic agents (Teicher *et al*, 1990; Thews *et al*, 2001) or photodynamic therapy (Henderson and Fingar, 1987). However, an increasing number of studies clearly demonstrate that tumour hypoxia also has an impact on gene expression, with changes in glycolytic enzymes, growth factors or angiogenic molecules (for a recent review, see Höckel and Vaupel, 2001). Many of these genes are under the control of the hypoxia-inducible factor 1 (HIF-1), which has been found to be accumulated in many tumours (Semenza, 2000, 2002). Most of these processes of gene induction are not tumour specific and also take place in the normal tissue during hypoxia. However, some of them (e.g. hypoxia-induced neovascularisation) may contribute to malignant progression and metastasis (Dachs and Tozer, 2000). In particular, the formation of new blood vessels in growing tumours, which is stimulated by hypoxia-induced upregulation of the vascular endothelial growth factor (VEGF) (Sutherland *et al*, 1996), seems to play an important role in malignant progression. Folkman (1971, 1995) postulated that tumour growth to macroscopic size only becomes possible as a result of this neovascularisation induced by secretion of angiogenic factors, as a result of which the improved tumour blood flow ensures the supply of oxygen and nutrients necessary for further cell proliferation (Harris, 2002). Thus, tissue hypoxia seems to be important for a needs-related modification of the oxygen and nutrient supply, the pH or the bioenergetic status, which together form the so-called metabolic tumour microenvironment. Hypoxia may therefore lead to an improvement in the oxygenation status of the tumour and thus promote subsequent tumour progression (Coleman *et al*, 2001). However, besides hypoxia, other mechanisms (e.g. oncogene expression) also seem to contribute to angiogenesis (Feldkamp *et al*, 1999; Zhang *et al*, 2001; Rak and Yu, 2004).

Vessels formed during hypoxia-induced angiogenesis show several structural and functional abnormalities compared to those in normal tissues. These abnormalities include blind endings of vessels, irregular branching patterns, loss of vascular hierarchy, absence of endothelial lining, increased vascular permeability and high fraction of arterio-venous shunt perfusion (Konerding *et al*, 1989a, 1989b, 1999; Vaupel *et al*, 1989, 2001), and may result in an inadequate perfusion, which does not follow a regular pattern (Vaupel *et al*, 1989). These abnormalities lead to increased intervascular distances limiting the diffusive oxygen flux to the tumour cells and thus result in an insufficient cellular O_2_ supply. In addition, tumour blood flow in these newly formed vessels has been seen to undergo temporal fluctuations with intermittent flow stops in individual vessels (Chaplin and Hill, 1995; Kimura *et al*, 1996).

From these findings it becomes clear that although hypoxia can stimulate angiogenesis and by this may increase blood supply to the tumour, it can at the same time cause limitations in the oxygen supply due to the structural abnormalities of the newly formed vessels. For this reason, the impact of long-term hypoxia on changes in the vascular structure, perfusion or metabolic microenvironment cannot be predicted. Due to hypoxia-induced changes in the activities of glycolytic enzymes, the metabolic behaviour of the tumour may change so that chronic hypoxia may also induce fundamental changes in the microenvironment. These aspects become even more important considering that the aim of some therapeutic concepts is to increase tumour hypoxia (Wood *et al*, 1994) in order to improve the efficacy of anticancer drugs, which act preferentially on hypoxic cells (Coleman *et al*, 1984; Brown, 1993; Greco *et al*, 2002; Wouters *et al*, 2002; Henk *et al*, 2003). At present, it is unclear what impact long-term hypoxia has on the microenvironment of solid tumours. In particular, the question arises whether chronic tumour hypoxia (over several days) induces adaptive processes modulating the oxygen and nutrient supply to the cells.

The aim of the present study was to assess changes in oxygenation and metabolic and bioenergetic parameters as a consequence of an increase in tumour hypoxia. The O_2_ status of experimental tumours was therefore intentionally worsened by inspiratory hypoxia over the whole period of tumour growth (6–14 days). Subsequently, oxygenation, metabolic and bioenergetic parameters of the tumours were determined. In order to study the underlying mechanisms by which the oxygen and nutrient supply of the tissue was modified, vascularisation and perfusion were evaluated.

## MATERIALS AND METHODS

### Animals and tumours

Male Sprague–Dawley rats (Charles River Deutschland, Sulzfeld, Germany; body weight 190–240 g) housed in our animal care facility were used in this study. They received a standard diet and acidified water *ad libitum*. Experimental tumours grew following subcutaneous injection of DS-ascites tumour cells (0.4 ml; approximately 10^4^ cells *μ*l^−1^) into the dorsum of the hind foot, as flat, spherical segments and replaced the subcutis and corium completely. The volume was determined by measurement of the three orthogonal diameters of the tumour and using an ellipsoid approximation with the formula *V*=*d*_1_*d*_2_*d*_3_*π*/6. Tumour volume doubling time was calculated from growth curves during the exponential growth phase (days 4–12 after inoculation). Microenvironmental measurements were performed when tumours reached a target volume of 0.5–3.0 ml approximately 6–14 days after inoculation. Animals were housed either under normoxic ambient conditions (room air; 21% O_2_) or in a hypoxic atmosphere containing 8% O_2_ (*chronic* hypoxia) continuously for the whole period of tumour growth and during measurements (e.g. oxygenation measurements). For experiments analysing the impact of *acute* hypoxia, animals were housed under normoxic conditions during tumour growth but breathed the hypoxic gas mixture (8% O_2_) for 20 min prior to and during the measurements. Studies had previously been approved by the regional ethics committee and were conducted according to UKCCCR guidelines (Workman *et al*, 1998) and the German Law for Animal Protection.

### Tumour oxygen tensions

The distribution of tumour oxygen tensions (oxygen partial pressure, *p*O_2_) was measured polarographically using steel-shafted microelectrodes (outer diameter: 300 *μ*m) and the *p*O_2_ histography system (Eppendorf, Hamburg, Germany; for more details of this method, see Vaupel *et al*, 1991). A small midline incision was made in the skin covering the lower abdomen and the Ag/AgCl reference electrode was placed between the skin and the underlying musculature. For tumour *p*O_2_ measurement, a small incision was made in the skin overlying the tumour using a 24-gauge needle and the electrode advanced to a depth of approximately 1 mm. The electrode was then automatically moved through the tissue in preset steps with an effective step length of 0.7 mm. Approximately 100 *p*O_2_ values were obtained from each tumour in up to eight parallel electrode tracks. The oxygenation status of each tumour was described by the mean and median *p*O_2_ as well as by the fraction of *p*O_2_ values ⩽2.5 and ⩽5 mmHg. Oxygenation studies of individual tumours were generally carried out in less than 20 min. Additionally, arterial blood gas analysis was performed immediately before and after tumour tissue *p*O_2_ measurements using a pH/blood gas analyser (type ABL 5, Radiometer, Copenhagen, Denmark) to ensure that values for the arterial blood gases were within the physiological range during the measurement period. Mean arterial blood pressure (MABP) was continuously monitored through connection of an arterial catheter placed in the left carotid artery to a Statham pressure transducer (type P 23 ID, Gould, Oxnard, CA, USA).

### Metabolite concentrations

In order to analyse metabolic parameters, the tumour-bearing hind limbs of the anaesthetised animals were rapidly frozen in liquid nitrogen. Tumours were subsequently removed, ground to a fine powder and freeze-dried. Thereafter, glucose and lactate concentrations were assayed enzymatically using standard test kits (#1442457 and #256773; Boehringer-Mannheim, Mannheim, Germany), and ATP, ADP and AMP were measured by HPLC (for details of the methods, see Vaupel *et al*, 1994; Kelleher *et al*, 1995).

### Vascular endothelial growth factor concentration

Vascular endothelial growth factor was determined by an ELISA kit detecting rat VEGF (kit DY564, R&D Systems, Minneapolis, MN, USA) according to the manufacturer's instructions. In brief, tumours were excised and cut into small pieces. Approximately 0.5 g of tissue was suspended in 1.5 ml RIPA buffer (phosphate-buffered saline, NP-40, Na^+^-deoxycholate+proteinase inhibitor cocktail), sonicated five times on ice and centrifuged. The protein content of the supernatant was determined using Bradford reagent (Sigma, Deisenhofen, Germany). For each measurement, approximately 50 *μ*g of protein was analysed. For this, 96-well microplates were coated with a mouse anti-rat VEGF antibody. The supernatant of the tissue preparation was diluted in PBS+1% BSA (1 : 10 v v^−1^) and incubated in the wells for 2 h at room temperature. After washing, the detection antibody (goat anti-rat VEGF) was added and incubated for a further 2 h at room temperature. The optical density was determined on a microplate reader at a wavelength of 450 nm. The VEGF concentration determined for each well was normalised to the protein content in the sample. In contrast to all other experiments, in the VEGF study, the animals in the group ‘acute hypoxia’ were exposed to the hypoxic atmosphere (8% O_2_) for 18 h (not just for 20 min) prior to tumour excision in order to leave sufficient time for the *in vivo* upregulation of VEGF expression to take place.

### Vascular density

Endothelial cells were stained with a CD31 antibody. Cryosections with a thickness of 7 *μ*m were fixed for 15 min in acetone (4°C). Sections were rinsed in PBS (pH 7.4) and thereafter with 3% H_2_O_2_. Sections were washed in PBS between all the following steps. The specimens were incubated in normal horse serum (Vector Laboratories, Burlingame, CA, USA) for 20 min and incubated overnight in CD31 antibody (1 : 100, mouse anti-rat antibody MCA1334G, Serotec, Oxford, UK) at room temperature in humidified chambers. Biotinylated anti-mouse IgG (dilution 1 : 200, BA-2000, Vector Laboratories) was applied as a secondary antibody for 30 min, followed by ABC complex (peroxidase standard kit, PK 4000, Vector Laboratories) for 30 min. Immune complexes were made visible by incubation with AEC chromogene (peroxidase substrate kit, AEC SK-4200, Vector Laboratories) for 15 min. The specimens were then counterstained with haematoxylin (1 : 2) for 2 min and rinsed in tap water. Finally, using aquatex mounting medium (Merck, Darmstadt, Germany), specimens were mounted with a coverslip.

All morphometric analyses were performed single-blinded by the same observer using a computerised digital image analysis system (Diskus 4.30, Hilgers, Königswinter, Germany) connected with a Zeiss Axiophot microscope (Zeiss, Jena, Germany). In each tumour, the areas with the highest microvessel density (hot spots) were identified. Quantification of the microvessels was performed in five hot spots in the tumour periphery and five hot spots in the tumour centre. The vessel counts were averaged separately for the tumour centre and periphery. Analysis of the fraction of viable tumour tissue was performed in the haematoxylin and eosin-stained slices by marking necrotic areas and calculating the fraction of necrosis in the image.

### Perfusion-related parameters

In order to assess parameters related to the perfusion distribution within a tumour, the fluorescent dye Hoechst 33342 (Sigma, Deisenhofen, Germany) was injected i.v. at a dose of 25 mg kg^−1^ body weight. For this, the dye was dissolved at a concentration of 10 mg ml^−1^ in isotonic saline and approximately 0.5 ml of this stock solution was rapidly injected into a tail vein. At 60 s after injection, animals were killed by an overdose of anaesthetic and the tumours were rapidly frozen in liquid nitrogen. Following subsequent tumour removal, cryosections were prepared. Using fluorescence microscopy (filter set #02, Zeiss, Jena, Germany, excitation wavelength: 365 nm, emission filter: low-pass 420 nm) at low resolution (range of vision 3 × 2.5 mm), images of perfused vessels were taken from two to three different regions of each cryosection. Approximately three to four sections from each tumour were analysed resulting in 9–10 images per tumour. In these images, each vessel was marked and the mean density of perfused vessels, the mean distance between neighbouring vessels and the area of vascular domains were calculated using image analysis software (Optimas Ver. 6.2, Media Cybernetics, Silver Spring, MD, USA).

### DNA content and proliferation analysis

For analysis of DNA content and the fraction of actively DNA-synthesising cells, tumours were labelled with bromodeoxyuridine (BrdU). For this, BrdU (Boehringer-Mannheim, Mannheim, Germany) was dissolved in PBS at a concentration of 10 mg ml^−1^ and injected i.p. (150 mg kg^−1^ body weight). After 120 min, tumours were excised and dissipated into a single cell suspension. Incorporated BrdU was detected using monoclonal antibodies and DNA content was determined by propidium iodide staining (for details of the methods, see Thews *et al*, 1996).

### Blood cell parameters

Blood cell parameters were assessed using a multiparameter, automated haematology analyser (Ac-T8; Beckman-Coulter, Krefeld, Germany), whereby erythrocyte, white blood cell and platelet counts together with the mean red blood cell (RBC) volume (MCV) were measured by impedance and the haemoglobin concentration was measured by a photometric method. In addition, the analyser uses the measured values to calculate several other parameters (e.g. haematocrit, mean corpuscular haemoglobin content (MCH)). All measurements were performed using a sample of venous blood (20 *μ*l) taken from a tail vein.

### Statistical analysis

Results are expressed as means±s.e.m. unless stated otherwise. Differences between groups were assessed by the two-tailed Wilcoxon test for unpaired samples. The significance level was set at *α*=5% for all comparisons.

## RESULTS

Exposing rats to an atmosphere containing a reduced oxygen fraction (8%) over a prolonged period of time (6–14 days) affects several systemic parameters. As might be expected, inspiratory hypoxia markedly stimulated erythropoiesis, as indicated by a significantly higher haemoglobin concentration and haematocrit as well as an increased number of RBCs (Table 1

**Table 1:** RBC-related parameters in control animals and animals housed under chronically hypoxic conditions (inspiratory O_2_ fraction=8%) for the whole period of tumour growth (6–14 days)

	**Control**	**Chronic hypoxia**	***P*-value**
*n*	7	6	
cHb (g l^−1^)	137±4	167±3	0.003
Haematocrit (%)	42±1	51±1	0.003
RBC count (10^6^ *μ*l^−1^)	7.0±0.2	8.4±0.1	0.005
Mean corpuscular volume (MCV) (fl)	59±1	61±1	0.284
Mean corpuscular haemoglobin (MCH) (pg)	20±1	20±1	0.316

*n*=number of tumours investigated; cHb=haemoglobin concentration.

). However, mean erythrocyte volume (MCV) and cellular haemoglobin content (MHC) remained in the normal range. In parallel, the spleen wet weight of the animals was significantly reduced under hypoxic conditions (0.82±0.23 g) compared to control animals (1.06±0.24 g, *P*=0.032). Hypoxia led to a significant reduction in arterial *p*O_2_ followed by a stimulation of ventilation as indicated by a lower arterial *p*CO_2_ and a higher pH (Table 2

**Table 2:** Arterial *p*O_2_, *p*CO_2_ and pH in control animals, in animals during acute reduction of the inspiratory O_2_ fraction for 20 min (acute hypoxia) and during chronic inspiratory hypoxia for 6–14 days (chronic hypoxia)

	***n***	***p*O_2_ (mmHg)**	***p*CO_2_ (mmHg)**	**pH**
Control	19	78±1	48±1	7.41±0.03
Acute hypoxia	22	33±2*	32±1*	7.50±0.01*
Chronic hypoxia	17	38±1*, **	29±2*	7.49±0.01*

*n*=number of tumours investigated.

* *P*<0.001 *vs* control;

** *P*<0.05 *vs* acute hypoxia.

). Arterial *p*O_2_ in chronically hypoxic animals was slightly higher compared to acute hypoxia (38±1 *vs* 33±2 mmHg), possibly indicating an adaptation of ventilation to a chronically reduced inspiratory O_2_ fraction.

**Figure 1:**
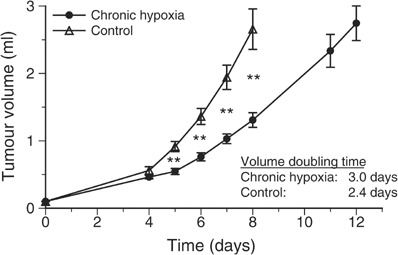
Tumour growth during chronic inspiratory hypoxia (O_2_ fraction 8%, *n*=22) and in normoxic control tumours (*n*=18). Values are expressed by mean±s.e.m.; ^**^P<0.001.

Housing animals under hypoxic environmental conditions also had an impact on the growth behaviour of experimental tumours. Under control conditions (breathing room air), the DS-sarcoma used in the study had a volume doubling time of 2.4 days (during the exponential growing phase), whereas tumours growing under inspiratory hypoxia had a significantly longer volume doubling time (3.0 days; Figure 1

). Since the dependency of oxygenation, bioenergetic status, fraction of viable tissue and perfusion on tumour volume is well documented for many experimental tumour models, all comparisons of these parameters in this study were performed on tumours of comparable size. For this reason, parameters were measured on different days after tumour implantation when tumours reached a mean volume of approximately 1.5 ml.

**Figure 2:**
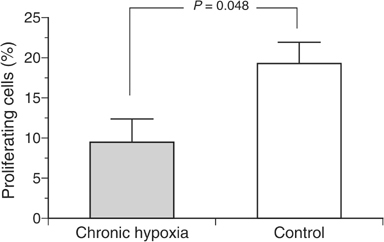
Fraction of actively DNA-synthesising cells as measured by BrdU incorporation during chronic inspiratory hypoxia (*n*=8) and under control conditions (*n*=18).

Although the cell cycle distribution was not markedly different between the two groups, the fraction of actively DNA-synthesising cells as measured by BrdU incorporation was significantly lower in the group exposed to inspiratory hypoxia (Figure 2

). Since the fraction of cells in the S phase of the cell cycle was not different, the fraction of cells not incorporating BrdU during the S phase was slightly higher in chronically hypoxic cells than under control conditions. These data indicate a prolongation of the cell cycle S phase during chronic hypoxia rather than an arrest of cells during proliferation.

**Figure 3:**
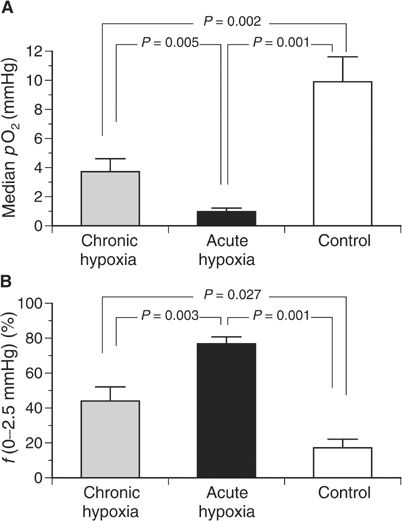
(**A**) Median tumour *p*O_2_ and (**B**) fraction of hypoxic *p*O_2_ values ⩽2.5 mmHg during chronic inspiratory hypoxia (chronic hypoxia, *n*=17), acute reduction of the inspiratory O_2_ fraction for 20 min (acute hypoxia, *n*=18) and under normoxic control conditions (*n*=21).

Inspiratory hypoxia strongly affects the oxygenation status of the tumour. An *acute* reduction in the inspiratory O_2_ fraction to 8% reduces the median *p*O_2_ from 10 mmHg (control) to 1 mmHg within 20 min (Figure 3

). In parallel, the fraction of hypoxic *p*O_2_ values ⩽2.5 mmHg significantly increased from 17 to 77% (Figure 3). However, during *chronic* hypoxia (lasting the whole period of tumour growth), the worsening of the O_2_ status was less pronounced: the median *p*O_2_ was 4 mmHg and the fraction of hypoxic *p*O_2_ values ⩽2.5 mmHg was 44% (Figure 3).

**Figure 4:**
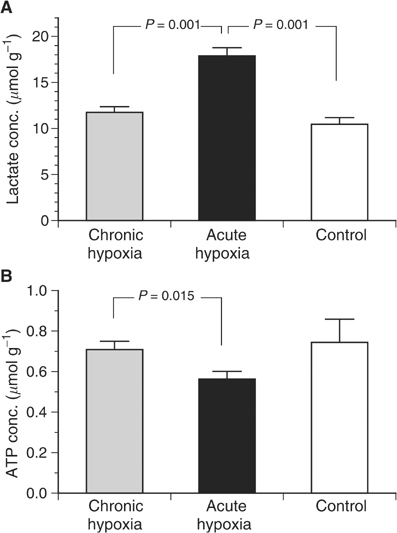
(**A**) Mean lactate and (**B**) ATP concentrations in tumours during chronic inspiratory hypoxia (chronic hypoxia, *n*=22), acute reduction of the inspiratory O_2_ fraction for 20 min (acute hypoxia, *n*=30) and under normoxic control conditions (*n*=12).

These differences in tumour oxygenation also have an impact on the metabolic and bioenergetic status. Although the glucose concentration in the tumours was not markedly different between the three groups (control: 1.35±0.13 *μ*mol g^−1^; acute hypoxia: 1.43±0.10 *μ*mol g^−1^; chronic hypoxia: 1.07±0.10 *μ*mol g^−1^), the lactate level in the group acutely exposed to inspiratory hypoxia was significantly higher compared to control animals and to chronically hypoxic tumours (Figure 4A

), presumably indicating an increased level of glycolysis. In turn, the ATP concentration in the acute hypoxia tumours was significantly lower compared to those in control animals and under chronic hypoxia (Figure 4B).

Although the results of tumour oxygenation and metabolic parameters indicate that the O_2_ supply and thus the energy yield are reduced during inspiratory hypoxia, the tumour tissue was still found to be non-necrotic. The fraction of viable tissue was 70±3% in control tumours and 75±6% in tumours grown under chronically hypoxic conditions. Even though apoptosis was not measured directly in this study, the fraction of tumour cells with a DNA content below that of G_0_ cells can be used as a measure of apoptosis. This fraction was found to be low in chronically hypoxic as well as in control tumours (9±1 and 5±1%, respectively; *P*=0.002).

**Figure 5:**
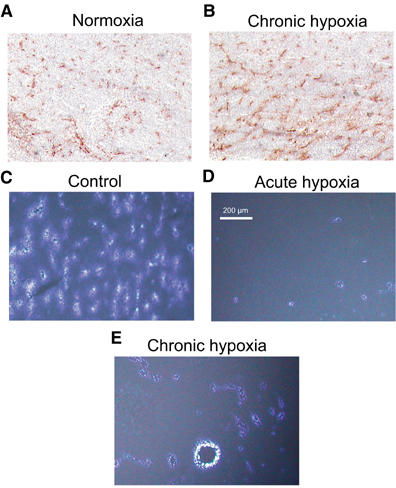
Examples of vascular patterns (CD31 staining) in tumours growing under either (**A**) normoxic conditions or (**B**) during chronic inspiratory hypoxia, and of perfusion distribution (Hoechst 33342 staining) under (**C**) normoxic control conditions, during (**D**) acute reduction of the inspiratory O_2_ fraction for 20 min or during (**E**) chronic inspiratory hypoxia. All images are scaled to the same magnification.

In addition, no marked differences were seen in the vascularity of tumours in both groups (Figure 5A and B

). In order to analyse the impact of inspiratory hypoxia (resulting in a pronounced O_2_ deficiency in the tumour) on the induction of vessel formation, the vascular densities in areas of high vascular density (hot spots) in the tumour centre and periphery were determined. However, neither the size of the hot spots nor the mean or maximum vascular density within the hot spots (Table 3

**Table 3:** Area size of vascular ‘hot spots’, mean and maximum vascular density within the ‘hot spots’ in control tumours and in tumours grown under chronically hypoxic conditions *in vivo* (inspiratory O_2_ fraction=8%)

	**Tumour centre**		**Tumour periphery**	
	**Control**	**Chronic hypoxia**	**Control**	**Chronic hypoxia**
*n*	6	10	6	10
Area of hot spots (mm^2^)	0.21±0.07	0.22±0.04	0.15±0.02	0.21±0.04
Mean vascular density in the hot spots (vessels mm^−2^)	348±49	258±40	315±55	322±63
Maximum vascular density in the hot spots (vessels mm^−2^)	529±62	385±53	507±101	423±80

**Figure 6:**
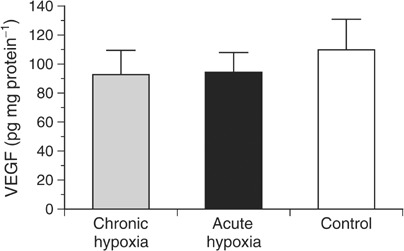
Vascular endothelial growth factor concentration in tumours during chronic inspiratory hypoxia (chronic hypoxia, *n*=4), acute reduction of the inspiratory O_2_ fraction for 18 h prior to VEGF measurements (acute hypoxia, *n*=4) and under normoxic control conditions (*n*=8).

Tumour areas both in the centre and periphery were analysed. *n*=number of tumours investigated.

) was markedly different (and not statistically significant) between the groups studied. The results of the vascularity evaluation reflect the findings of the VEGF concentration measurements. Even though the DS-sarcoma cells are able to upregulate VEGF expression under hypoxic conditions *in vitro* (data not shown), no marked differences in the VEGF level in tumours of the different groups were seen in the *in vivo* situation (Figure 6

). Higher VEGF concentrations as compared to controls were not found either in tumours of animals kept under hypoxia only for 18 h prior to tumour excision (‘acute hypoxia’) or in animals housed for the whole period of tumour growth in an oxygen-reduced atmosphere (‘chronic hypoxia’).

**Figure 7:**
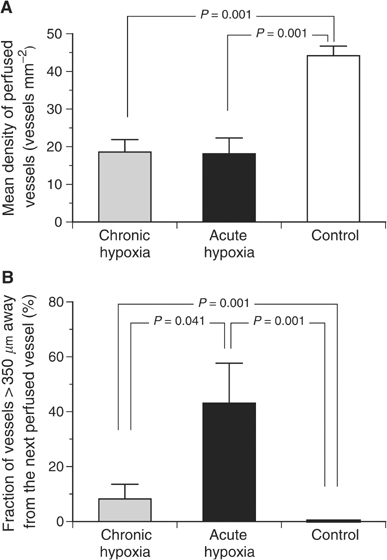
(**A**) Mean density of perfused vessels and (**B**) fraction of vessels more than 350 *μ*m away from the next perfused vessel (as determined by Hoechst 33342 injection) in cryosections of tumours during chronic inspiratory hypoxia (chronic hypoxia, *n*=12), acute reduction of the inspiratory O_2_ fraction for 20 min (acute hypoxia, *n*=14) and under normoxic control conditions (*n*=10). In all control tumours, the distances between neighbouring perfused vessels never exceeded 350 *μ*m.

Even though the number of vessels (as indicated by CD31 staining) was not different in chronically hypoxic tumours as compared to controls (Figure 5A and B), the pattern of perfusion distribution varied profoundly (Figure 5C–E). In acutely hypoxic tumours, the density of perfused vessels was more than halved as compared to control animals (Figures 5D and 7A

). In parallel, the mean distance between neighbouring perfused vessels was dramatically increased (655±181 *μ*m) to values far greater than the oxygen diffusion distance. In tumours under chronic inspiratory hypoxia conditions, the mean number of perfused vessels was also reduced to approximately the same extent as during acute hypoxia (Figure 7A). However, the spatial distribution of perfusion in these tumours was much less heterogeneous (Figure 5E). In chronically hypoxic tumours, the mean intervascular distance between perfused vessels was markedly reduced (317±114 *μ*m, although due to large interindividual variability it was not statistically significant). These data indicate a trend to a more uniform perfusion compared to acute hypoxia, although values of control tumours were not reached (134±3 *μ*m). This trend also becomes obvious when the fraction of vessels that are more than 350 *μ*m away from the next perfused vessel is calculated (Figure 7B). One possible reason for the reduced perfusion seen during inspiratory hypoxia despite a constant number of tumour vessels may be a decrease in MABP during hypoxia. In control animals, MABP was 139±2 mmHg, in acute hypoxia it was reduced to 77±2 mmHg and in animals kept chronically under hypoxia it was 88±3 mmHg (*P*=0.0052 *vs* acute hypoxia).

## DISCUSSION

In the present study, the impact of pronounced chronic hypoxia on adaptive processes in tumours has been analysed. For this, experimental tumours, which show O_2_ deficiency even under control conditions (approximately 20% *p*O_2_ values below 2.5 mmHg), were artificially made even more hypoxic by exposing the animals to a hypoxic atmosphere containing 8% oxygen for the period of tumour growth. However, this procedure induces severe hypoxia systemically in the whole animal rather than just in the tumour. Therefore, several other processes that are not directly related to tumour hypoxia and that in turn may affect the tumour microenvironment might be induced. For instance, the measured blood gas parameters (Table 2) indicate that during acute as well as during chronic inspiratory hypoxia, ventilation was stimulated and a respiratory alkalosis was induced leading to a higher affinity of O_2_ to haemoglobin (as indicated by a left shift of oxyhaemoglobin dissociation curve). This mechanism may contribute to the worsening of the O_2_ supply to the tumour and by this increase tumour hypoxia. On the other hand, hypoxaemia induces erythropoietin (EPO) formation in the kidney resulting in forced erythropoiesis (as was the case in the present study; Table 1). Since several tumour cell lines express an EPO receptor (Acs *et al*, 2001), it has been discussed that erythropoietin may promote tumour growth (Acs *et al*, 2003). However, for the tumour line used in the present study (DS-sarcomas of the rat), it has been shown that EPO *per se* has no promoting effect on proliferation (Thews *et al*, 1998). On the other hand, secondary indirect effects of EPO on the tumour microenvironment (e.g. by increasing the haemoglobin level of the blood and by this improving the oxygen transport capacity; Table 1) cannot be ruled out in the present study. Therefore, the results obtained from the artificial situation of systemic hypoxia can only be transferred with caution to the situation found clinically in tumour patients.

The oxygen deficiency seen in many experimental and human tumours results from a discrepancy between oxygen demand and supply to the tissue. In particular, the convective oxygen transport with the blood is insufficient since the tumour vasculature shows chaotic vessel structure (Konerding *et al*, 1989a, 1989b, 1999) resulting in a loss of coordinated blood flow (Vaupel *et al*, 1989; Secomb *et al*, 1995). In addition, temporary stasis of blood flow has been described in many experimental and human tumours (Chaplin and Hill, 1995). During systemic hypoxia, tumour blood flow becomes even worse whereby pronounced differences in the perfusion pattern of tumours were seen between *acute* and *chronic* hypoxia. During short-term hypoxia, the number of perfused vessels was dramatically decreased (Figures 5D and 7A), which may be the result of a hypoxia-induced reduction in arterial blood pressure (normoxia: 139±2 mmHg; acute hypoxia: 77±2 mmHg). Such a decrease, which has also been described by others (Marshall and Metcalfe, 1989; Sato *et al*, 1992; Trzebski *et al*, 1995) may have several reasons. Inspiratory hypoxia has been shown to induce a more pronounced formation of NO in the lung, which may then lead to a systemic vasodilation resulting in a reduction of perfusion pressure (Hubloue *et al*, 2003). It has also been demonstrated that hypoxaemia can directly induce vasodilation in various organs (e.g. brain, skeletal muscle) (Marshall and Metcalfe, 1989; Harrison *et al*, 1990; Sato *et al*, 1992). However, Harrison *et al* (1990) showed that in skeletal muscle hypoxaemia presumably leads to an increase of perfusion of larger arteries whereas capillary blood flow was only marginally improved indicating a hypoxaemia-induced higher fraction of shunt perfusion. Such a redistribution of blood flow may also play a part in the present study in which pronounced systemic hypoxaemia is induced. Vasodilation in muscles of the hind limb may redistribute blood flow to the disadvantage of the tumour resulting in a reduction of perfusion disproportionate to the changes in perfusion pressure (‘steal phenomenon’) (Hirst, 1989). Another reason for this reduction in local perfusion could be a vasoconstriction of vessels feeding the tumour. However, since the tumours were implanted into the subcutis, this explanation seems unlikely since a hypoxic vasoconstriction has only been described in lung tissue.

Perfusion during acute inspiratory hypoxia was distributed very heterogeneously as indicated by a pronounced increase in the distance between neighbouring perfused vessels (Figures 5E and 7B) with a large intratumoral variability of this parameter. Tumour regions with an almost normal perfusion distribution were found closely adjacent to large areas of viable tumour tissue with almost no perfusion. During chronic hypoxia, the mean number of perfused vessels was not markedly changed as compared to acute hypoxia. However, in chronic hypoxia, perfusion was distributed much more homogeneously throughout the tissue as indicated by a significant decrease in the distance between neighbouring perfused microvessels (Figure 7B) with a smaller intraindividual variability.

One major consequence of the impaired tumour perfusion occurring during systemic hypoxia is a worsening of convective oxygen transport to the tissue. Due to the pronounced heterogeneity of tumour perfusion in *acutely* hypoxic tumours the median tissue *p*O_2_ is markedly reduced, whereas during *chronic* hypoxia the perfusion pattern becomes more homogeneous leading to an improvement of tumour oxygenation (Figure 3). However, in addition to the changes in the pattern of tumour perfusion, other reasons for a long-term adaptation to chronic hypoxia have to be considered. As described earlier (Tannock and Steel, 1970; Amellem and Pettersen, 1991), induction of chronic hypoxia reduced tumour cell proliferation as indicated by a slower tumour growth and a lower fraction of actively DNA-synthesising cells (Figures 1 and 2). This reduction in cell proliferation was not seen in anaemic animals using the same tumour model (Thews *et al*, 2001), presumably because anaemia resulted in an increase of tumour hypoxia, which is less pronounced (Kelleher *et al*, 1996) than that seen when animals were subjected to an atmosphere containing only 8% oxygen. Even though the proliferation rate was reduced in the present study, a cell cycle arrest in the G_1_ phase, as has been described in cell culture experiments (Fujii *et al*, 2002), was not found in the *in vivo* situation in the present study. The fraction of cells in the G_0_/G_1_ phase in tumours under hypoxic conditions was not significantly increased as compared to controls breathing room air. For this reason, the differences in proliferation and tumour growth rate found *in vivo* in the present study appear to be attributable to a prolongation of the cell cycle rather than a cell cycle arrest. Another explanation may be an alteration of the G_1_/S checkpoint, which has been described in several tumours. However, for the tumour model used in the present study (experimental DS-sarcoma of the rat), the status of this checkpoint is not known. For this reason, it might be possible that the lack of hypoxia-induced cell cycle arrest results from a deficient G_1_/S checkpoint. Since the oxygenation status of a tissue results from a dynamic steady state between O_2_ delivery and consumption, the reduced number of proliferating cells may also contribute to the better oxygenation under these conditions (Thews *et al*, 1999). However, a previous study showed that inhibition of DNA synthesis does not necessarily result in a decrease in the O_2_ consumption rate of tumour cells *in vivo* (Thews *et al*, 1996).

Hirst *et al* (1987a, 1991) developed a model of adaptation of tumours to long-term anaemia. They described that anaemia-induced hypoxia acutely leads to a higher fraction of hypoxic cells in the tumour. When hypoxia persisted over several days, the cord of viable tumour cells around vessels was reduced, resulting in a hypoxic fraction comparable to that found in nonhypoxic controls (similar to the improvement of oxygenation in chronically compared to acutely hypoxic tumours of the present study). This model of adaptation is, however, not directly transferable to our findings described here. If a reduced O_2_ diffusion radius is proposed, the vascular density in these tumours should increase. In the present hypoxia model used, no differences in the morphological pattern of microvessels were found (in tumours of comparable size and with similar fractions of necrotic tissue regions). For this reason, other adaptive mechanisms have to take place in addition to the reduction of the O_2_ diffusion distance.

In the experimental setting used, improvement of tumour hypoxia during chronic inspiratory hypoxia may also be the result of the increased haemoglobin concentration seen (Table 1), which would lead to a higher oxygen transport capacity. Another mechanism in this context has been proposed by Hirst and Wood (1987a, 1987b) and involves an adaptation of tumours to long-term anaemia, again resulting in less pronounced tumour hypoxia. The authors showed that an increase in the level of 2,3-diphosphoglycerate (2,3-DPG) in erythrocytes during inspiratory hypoxia reduced the affinity of O_2_ to haemoglobin and by this improved the oxygen supply to the tumour cells. Even though 2,3-DPG levels were not determined in the present study, this mechanism may also contribute to the long-term improvement of the O_2_ status to chronic hypoxia. Finally, the arterial *p*O_2_ in chronically hypoxic animals was slightly higher than during acute hypoxia (Table 2), indicating an adaptation of ventilation to chronic reduction of inspiratory O_2_ fraction. This higher arterial *p*O_2_ may also (at least partially) contribute to the higher tumour *p*O_2_ observed.

Another mechanism of long-term adaptation of tumour cells as well as of host tissue cells (e.g. fibroblasts in the stroma) to hypoxic conditions has to be ruled out in the present study. It has been postulated that severe hypoxia may induce the formation of new tumour blood vessels necessary for further tumour growth (Folkman, 1995). In many human tumours, a close spatial correlation of local oxygen deficiency (determined by assessment of intrinsic or extrinsic hypoxia markers, eg, HIF-1*α* or EF5) and the production of VEGF was found (Damert *et al*, 1997; Maxwell *et al*, 1997; Blancher *et al*, 2000; Huss *et al*, 2001; Ziemer *et al*, 2001; Buchler *et al*, 2003). Pilch *et al* (2001) showed in an *in vitro* experiment that tumour cells – and even more so tumour stroma-derived fibroblasts – increase their VEGF secretion rate when they are kept under hypoxia (*p*O_2_ <1.5 mmHg for 24 h). Under these conditions, tumour cells were able to increase the VEGF production by up to 10-fold compared to the basal secretion found under normoxia (*p*O_2_=150 mmHg). It should be noted, however, that these results varied between the different cell lines used. Fibroblasts were able to increase VEGF secretion by a factor of four. DS-sarcoma cells used in the present study produce small quantities of VEGF even under normoxic conditions (approximately 24 pg h^−1^ 10^−6^ cells). When these cells were kept under hypoxia (*p*O_2_ <1.5 mmHg for 24 h), VEGF secretion increased by a factor of six indicating that, in the tumour cell line used in the present study, hypoxia is able to pronouncedly induce VEGF formation *in vitro*. However, *in vivo*, the VEGF concentration in the tumour was not markedly different in animals kept under chronic hypoxia compared to normoxic conditions (Figure 6). Since the HIF-1*α* level was not determined in the present study, it remains unclear whether hypoxia *in vivo* leads to HIF-1 accumulation (either directly or indirectly by increasing the NO concentration; Metzen *et al*, 2003) in the tumour model used. However, notable is the finding that pronounced systemic and tumour hypoxia was not able to increase VEGF concentrations markedly. As a result of these unchanged VEGF levels, parameters describing the vascular network (vascular density in tumour centre and periphery) showed no significant differences (Table 3).

Several explanations for the lack of VEGF induction followed by changes in the vascular structure *in vivo* as compared to cell culture experiments have to be taken into account. Since the DS-sarcomas show pronounced hypoxic regions with almost 50% of *p*O_2_ values below 2.5 mmHg (Thews *et al*, 1997), it may be that VEGF production is maximally stimulated even when animals are breathing room air, such that a further increase, due to a more hypoxic environment, might not be possible. Another possible reason for the lack of an increased VEGF induction may be that the tissue hypoxia induced by a reduction of the inspiratory O_2_ fraction is not strong enough. Under *in vitro* conditions, VEGF is induced following incubation at very low *p*O_2_ values (<1 mmHg) for several hours (Maity *et al*, 2001; Pilch *et al*, 2001). Even though the median tumour *p*O_2_ was markedly reduced in the present study by the reduction of the inspiratory O_2_ fraction to 8% (Figure 3), it is conceivable that the tissue *p*O_2_ does not reach values low enough to stimulate VEGF formation due to temporal fluctuations in the oxygenation (Chaplin *et al*, 1987).

Inspiratory hypoxia led to marked changes in the metabolic and bioenergetic status with a decreased glucose and ATP concentration and an increase in the lactate level (Figure 4). These findings could be the result of pronounced tumour hypoxia leading to an upregulation of enzymes of the glycolytic pathway leading to increased lactate concentration, an acidic extracellular pH and a reduced ATP yield seen in many tumours (Vaupel *et al*, 1994). However, these findings could also be the result of the observed reduction in tumour perfusion. In this case, the nutrient supply (glucose) as well as the lactate elimination from the tissue would be reduced. For this reason, the observed metabolic changes not only reflect cellular adaptation to chronic hypoxia but also mirror indirectly changes of tumour perfusion. Maybe, both mechanisms together are responsible for the observed metabolic changes.

In conclusion, pronounced long-term hypoxia in an experimental tumour induces adaptive processes resulting in an improvement of the oxygenation status (as compared to acute hypoxia). However, the increase in median *p*O_2_ does not result from a hypoxia-induced angiogenesis (e.g. by induction of VEGF) but from a functional adaptation of the perfusion pattern. Acute hypoxia results in pronounced heterogeneity of the tumour blood flow distribution, whereas chronic hypoxia leads to a more homogeneous pattern with significantly smaller distances between perfused vessels resulting in a better diffusive O_2_ supply to the cells. In the tumour model used, functional changes in physiological parameters appear to play a more important role in the adaptation to chronic hypoxia than hypoxia-induced alterations in VEGF expression and morphological changes of vasculature.
